# Perils of Prolonged Impaction of Oesophageal Foreign Bodies

**DOI:** 10.5402/2011/621682

**Published:** 2011-06-13

**Authors:** Saumitra Saha, Anandabrata Bose

**Affiliations:** ^1^Department of Gastrointestinal Surgery, Medica North Bengal Clinic, Siliguri, West Bengal 7344003, India; ^2^Department of Otorhinolaryngology/Head & Neck Surgery, Medica North Bengal Clinic, Siliguri, West Bengal 7344003, India

## Abstract

Ill-conceived effort at removal of impacted foreign bodies (FBs) in oesophagus vies with delay in removal as the causes of morbidity and mortality. Most oesophageal FBs are safely removed endoscopically when attempted early. However, large sharp FBs like dentures and meat bones can get deeply embedded in the wall with prolonged impaction or injudicious attempts at removal leading to life-threatening mediastinitis. Open surgery to access the oesophageal-impacted FB in such an event is hazardous. This report emphasizes the need for early site-specific surgical approaches that may be required, albeit rarely, for oesophageal-impacted FBs, where attempts at endoscopic removal have failed or complications have ensued.

## 1. Introduction

Accidentally ingested foreign body is a common emergency, presenting to the otolaryngologists, gastroenterologists, gastrointestinal surgeons, and thoracic surgeons. Over 90% of them pass uneventfully through the gastrointestinal tract [1]. Of those lodged in the oesophagus, most are removed uneventfully with an endoscope. We report three patients where endoscopic removal failed or was not attempted; the resulting delay posed considerable difficulty in treatment and necessitated three different surgical approaches to the oesophagus in the face of life-threatening infection.

## 2. Case Report

**Patient 1**

**Figure 1:**
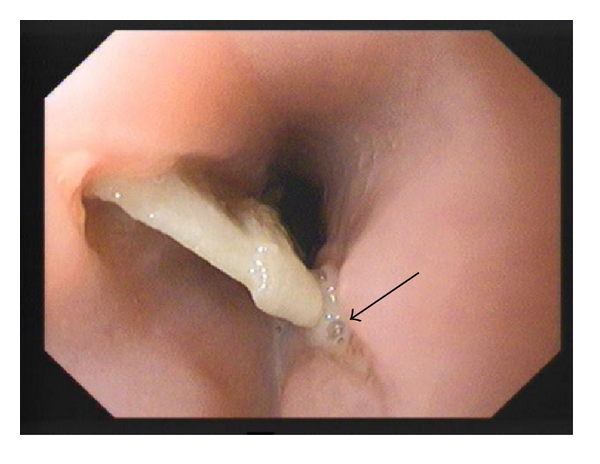
Videoendoscopy showing denture deeply embedded in oesophageal wall with sloughing of mucosa on the right lateral wall (arrow).

**Figure 2:**
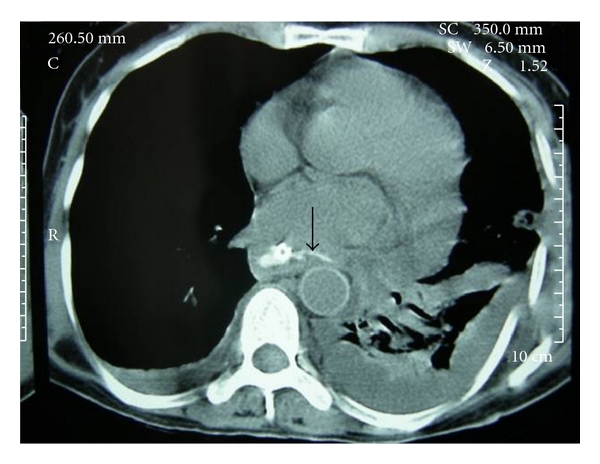
Leakage of oral contrast (arrow) from oesophagotomy repair site on CT scan.

A 60-year-old malnourished woman presented with dysphagia, fever, and retrosternal pain 7 days after accidental ingestion of a two-tooth denture with metal clasps. A chest X-ray (CXR) showed the metal clasps of the prosthesis in the region of the junction of mid and lower third oesophagus. Two attempts at flexible endoscopic removal elsewhere, 24 hours apart, was unsuccessful. A repeat videoendoscopy at our hospital showed an impacted denture at 29 cms (from the incisor teeth) with adjacent mucosal sloughing (Figure 1) which could not be dislodged. She was subjected to rigid oesophagoscopy under general anaesthesia (GA) which also failed to extract the denture. There was no option but to proceed to an urgent left thoracotomy through the 7th intercostal space. An oesophagotomy below the aortic arch revealed the denture with its hooks deeply embedded in an extremely thickened and oedematous oesophageal wall. The oesophagotomy was repaired using interrupted Vicryl sutures with a buttress of mediastinal pleura. A feeding jejunostomy was inserted (so that early enteral nutrition could be provided and continued in an event of leak), in addition to nasogastric tube and chest drains. On the 7th postoperative day, a CXR revealed moderate left pleural effusion, but a contrast X-ray on the 10th day did not show any leakage from the oesophagotomy repair site. However, oral methylene blue administration resulted in bluish discolouration of the basal chest drain effluent suggesting a leak and was confirmed on a contrast CT scan (Figure 2). She was fed through the jejunostomy for a further period of two weeks during which the leak resolved and oral feeding was gradually instituted.

**Patient 2**

**Figure 3:**
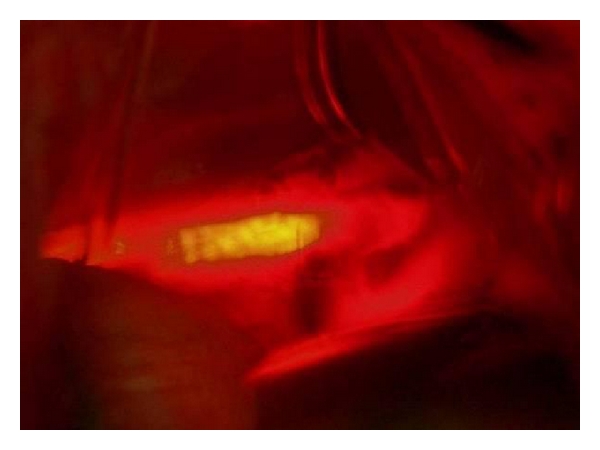
Intraoperative endoscopic transillumination to localize denture.

**Figure 4:**
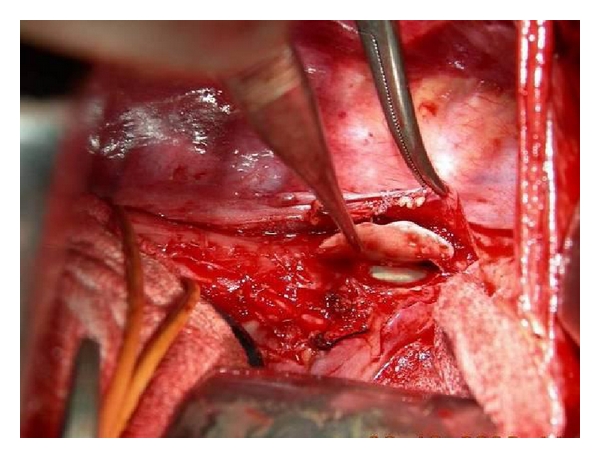
Longitudinal oesophagotomy to remove impacted denture.

A 65-year-old man accidentally swallowed a three-tooth denture (without clasps) at work away from his hometown. He developed dysphagia to solids. Flexible endoscopy 24 hours later noted the denture in lower oesophagus, but extraction had to be aborted after it got impacted at a more proximal level. His symptoms worsened, but he decided to travel to his hometown for further intervention, which delayed treatment for another 2 days. On presentation to us on the 5th day, he had severe chest pain, fever, and leukocytosis, but CXR was unremarkable. An urgent rigid oesophagoscopic extraction under GA failed as the denture was firmly impacted with one end appearing to have penetrated the oesophageal wall at 23 cms. The procedure was abandoned, and an urgent water soluble contrast X-ray was arranged. This showed mucosal irregularity in midoesophagus but no leakage. He underwent a posterolateral thoracotomy through the right 5th intercostal space. Severe mediastinitis causing extensive induration of the upper and midoesophagus with loss of tissue planes was encountered. Despite the large size of the denture, it could not be easily localized, and the site of oesophagotomy for its removal was determined by intraoperative flexible endoscopy. The silhouetted denture from endoscopic transillumination enabled a precise oesophagotomy (Figures 3 and 4). He resumed oral feeding after a normal contrast X-ray on the 10th postoperative day.

**Patient 3**

**Figure 5:**
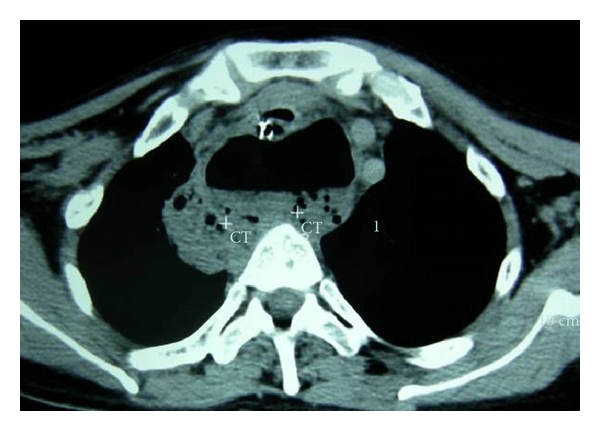
Large superior mediastinal abscess with multiple air pockets on CT scan.

A 26-year-old man accidentally ingested a meat bone. He ignored the slight dysphagia that followed, but 5 days later, had a bout of vomiting resulting in spontaneous expulsion of the meat bone. A few days later, he developed fever and neck pain for which he sought medical attention at the local hospital. A rigid oesophagoscopy under GA noted pus draining into the lumen from the retropharyngeal space. He was treated with intravenous antibiotics and fluids and nasogastric tube feeding over the next 7 days. The only imaging he underwent during this admission was a CXR which showed slight widening of the superior mediastinum. He was referred to our hospital 16 days after the accident, when he remained persistently febrile and developed neck tenderness. A repeat CXR showed retropharyngeal gas shadow besides a grossly widened superior mediastinum. A CT scan confirmed mediastinal abscess with air pockets (Figure 5). He proceeded to an urgent exploration through an oblique left neck incision, protecting the recurrent laryngeal nerve. The abscess was drained, but no perforation could be identified in the cervical oesophagus. The mediastinal drain was removed after 10 days. On the 15th day, he developed a localized subcutaneous neck abscess which was incised. This raised the possibility of an ongoing leak despite a normal oesophagogram. Oral feeding was instituted without problems after three weeks during which enteral nutrition was maintained through a nasogastric tube.

## 3. Discussion

Irregular oesophageal-impacted FBs mandate immediate extraction, and most are removed at fibreoptic or videoendoscopy [2]. A clear plan of action is required in the uncommon event of failure to extract the FB. Videoendoscopy is the first therapeutic modality attempted in our hospital if it has lodged beyond the cricopharyngeus. Radiolucent acrylic dentures can be localized by fluoroscopy after asking the patient to swallow a barium-soaked cotton wool which gets caught at the site of oesophageal impaction. Even if extraction at videoendoscopy fails, its magnified view accurately determines the degree of impaction which increases with passage of time [2]. A vigorous attempt at removal is not warranted in the presence of pus, slough, or bleeding, which indicates significant impaction or intramural perforation. Rigid oesophagoscopy under general anaesthesia should be the logical next step. However, disimpaction may be impossible even with the greater mechanical advantage of rigid tools particularly when treatment has been delayed. Specially designed shears to divide dental plates or bones into smaller pieces and aid its maneuverability have been described [3]. However, passage of such a robust shear through the rigid oesophagoscope may almost completely obliterate the lumen and the view, limiting its efficacy and safety. In both the patients where endoscopic removal failed, a thoracotomy had to be performed in the presence of inhospitable mediastinitis, perioesophageal induration with loss of natural tissue planes, and even requiring intraoperative flexible endoscopy in one patient to localize the FB. The tenuous vascularity due to prolonged impingement and inflammation makes oesophagotomy closure susceptible to leakage postoperatively and stricture formation in the long term, and it is imperative that a precise and short oesophagotomy is made. Both patients requiring thoracotomy had to be ventilated for short periods of 12–24 hours postoperatively to optimize oxygenation, adding to the costs of an already expensive and lengthy operation.

Injudicious and multiple attempts at flexible endoscopic removal of large dentures, particularly with metal clasps, may cause perforation as it is difficult to align the irregular-shaped denture favourably in relation to the lumen [4]. This implies that only an experienced endoscopist should attempt to remove these FBs. The exceptional course of spontaneous expulsion after a few days may herald delayed perforation from ischaemic necrosis as was seen in one patient. The open surgical access (cervical approach, right or left thoracotomy) to the oesophageal-impacted FB and their complications must be individualized and guided by endoscopic findings (distance of FB from incisor teeth) and imaging [5, 6]. While flexible endoscopy is widely available, the expertise of rigid oesophagoscopy, cervical exploration, thoracotomy, oesophageal surgery, and intensive care is often not available in a single unit or a peripheral hospital. The stand-alone endoscopist should therefore be cautious in attempting removal of such FBs. It would be prudent to refer these patients early to centres where prompt multidisciplinary input from the otolaryngologist/head and neck surgeon and oesophageal/thoracic surgeon is available. This is especially so when prolonged impaction beyond 12 hours decreases the chances of endoscopic removal and increases the hazards of open surgical removal.
